# Exploring the miRNA-mRNA Regulatory Network in Clear Cell Renal Cell Carcinomas by Next-Generation Sequencing Expression Profiles

**DOI:** 10.1155/2014/948408

**Published:** 2014-05-22

**Authors:** Sören Müller, Katharina Nowak

**Affiliations:** ^1^Plant Molecular Biology, Molecular BioSciences, Goethe University, Marie-Curie-Street 9, 60439 Frankfurt, Germany; ^2^GenXpro GmbH, Frankfurt Biotechnology Innovation Center, Altenhöferallee 3, 60438 Frankfurt, Germany; ^3^Department of Legal Science, Goethe University, Grüneburgplatz 1, 60323 Frankfurt, Germany

## Abstract

Altered microRNA (miRNA) expression is a hallmark of many cancer types. The combined analysis of miRNA and messenger RNA (mRNA) expression profiles is crucial to identifying links between deregulated miRNAs and oncogenic pathways. Therefore, we investigated the small non-coding (snc) transcriptomes of nine clear cell renal cell carcinomas (ccRCCs) and adjacent normal tissues for alterations in miRNA expression using a publicly available small RNA-Sequencing (sRNA-Seq) raw-dataset. We constructed a network of deregulated miRNAs and a set of differentially expressed genes publicly available from an independent study to *in silico* determine miRNAs that contribute to clear cell renal cell carcinogenesis. From a total of 1,672 sncRNAs, 61 were differentially expressed across all ccRCC tissue samples. Several with known implications in ccRCC development, like the upregulated miR-21-5p, miR-142-5p, as well as the downregulated miR-106a-5p, miR-135a-5p, or miR-206. Additionally, novel promising candidates like miR-3065, which *i.a.* targets *NRP2* and *FLT1*, were detected in this study. Interaction network analysis revealed pivotal roles for miR-106a-5p, whose loss might contribute to the upregulation of 49 target mRNAs, miR-135a-5p (32 targets), miR-206 (28 targets), miR-363-3p (22 targets), and miR-216b (13 targets). Among these targets are the angiogenesis, metastasis, and motility promoting oncogenes *c-MET*, *VEGFA*, *NRP2*, and *FLT1*, the latter two coding for VEGFA receptors.

## 1. Introduction

Approximately 3% of all cancers in adults occur in the kidney; therefore kidney cancer is one of the ten most frequently occurring cancers in western communities [1]. The primary histomorphologic type is clear cell renal cell carcinoma (ccRCC), which accounts for 80–85% of all kidney cancers followed by papillary renal cell carcinoma (pRCC) representing approximately 10% of renal cancers [2]. The gender and age distributions are similar between ccRCC and pRCC; however ccRCC has a worse prognosis with a 5-year survival of 77% [3, 4]. This is attributed to an advanced tumor stage (38.1%) and visceral metastasis at diagnosis (14.5%) [5].

MicroRNAs (miRNAs) are a class of small non-coding RNAs (sncRNAs) that can repress gene expression through translational repression or messenger RNA (mRNA) deadenylation and decay by base pairing to partially complementary sites [6]. Deregulated miRNAs have* i.a.* been associated with formation of metastases, tumor progression, and tumor growth in ccRCC and anti-miRs were suggested as novel therapeutic strategies in the treatment of the disease [7–9].

Next-generation small RNA-Sequencing (sRNA-Seq) allows for unbiased quantitative and qualitative sncRNA profiling. When compared to miRNA array platforms, sRNA-Seq additionally enables the discovery of novel miRNAs as well as the detection of other differentially expressed sncRNAs like small nucleolar RNAs (snoRNAs) and transfer RNA (tRNA)-derived fragments that can mimic miRNA function [10].

Several microarray based studies have demonstrated 21 to 34 differentially expressed miRNAs between ccRCC and normal kidney tissue [11]. SRNA-Seq studies reported more than 100 differentially regulated miRNAs, some of which might serve as diagnostic and prognostic markers [12, 13]. Nevertheless, these studies lack detailed information about miRNA targets and bioinformatical analysis is often only focused on miRNAs currently known to miRbase.

Here we used omiRas [14] to analyze a publicly available dataset (GEO: GSE24457) published by Zhou et al. [13], comprising twenty sRNA-Seq libraries of ten ccRCCs and ten adjacent control tissues from the same patient in order to identify sncRNAs with deregualted expression across all cases. After outlier detection with principle component analysis (PCA) samples of nine patients were used for downstream analysis.

We detected 61 sncRNAs as differentially expressed between the groups. Among these were several miRNAs without previous implication in kidney cancer development, like miR-3065-5p. Additionally, we detected seven snoRNAs and two tRNA derived fragments as differentially expressed between ccRCC and control tissues. We connected the deregulated miRNAs to biological pathways composed of differentially expressed genes under potential post-transcriptional control of these miRNAs. To do so, we utilized another publicly available mRNA-Sequencing (RNA-Seq) dataset (see methods). The “interaction network tool” of omiRas allows for the construction of interaction networks of miRNAs and mRNAs, interrogating the information from several miRNA-mRNA interaction databases. Therefore, we* in silico* assigned functions to significantly deregulated miRNAs and defined miRNAs implicated in the carciogenesis of ccRCC.

Among these is miR-206, which is significantly downregulated in ccRCC. Loss of miR-206 has been associated with hypoxia and under insufficient oxygen supply, angiogenesis is stimulated through upregulation of* VEGF* [15]. Our analysis revealed that miR-206 can regulate the expression of* VEGF* and several other genes involved in invasion, metastasis, and angiogenesis (*MET*,* FN1*,* NRP1*,* ELMO1*, and* TAGLN2*). This underlines that hypoxia induced loss of miR-206 expression is a critical process in clear cell renal cell carcinogenesis and maintenance.

Overall, our study demonstrates a promising strategy to identify driver miRNAs in cancer development that can afterwards undergo further functional testing.

## 2. Methods

### 2.1. Dataset Collection

A publicly available sRNA-Seq expression dataset of ten ccRCC and matching normal renal tissue was downloaded from the Gene Expression Omnibus (GEO: GSE24457) database in SRA format. A list of 1,299 significantly upregulated and 1,194 downregulated genes identified in mRNA-Seq data of 65 ccRCC cases from the Cancer Genome Atlas (TCGA) by Wozniak et al. [16] was retrieved in XLS format.

### 2.2. Data Preprocessing

Raw sequencing files were converted to FASTQ format and the 3′ sequencing adapter (*TCGTATGCCGTCTTCTGCTTGAAA*) was removed from the reads with cutadapt [17]. Subsequently, low quality stretches below a SANGER quality score of 20 were additionally trimmed from each end of the reads (*-q 20*). Only reads with a minimum length of 15 bps after clipping were used for further analysis (*-m 15*).

### 2.3. Data Analysis

#### 2.3.1. MiRNA Quantification, Outlier Detection, and Differential Expression Analysis

Preprocessed FASTQ-files were submitted to omiRas. Briefly, in omiRas, reads are summarized to UniTags. Singletons are removed from the data set and the remaining tags are mapped to the human genome (hg19) with bowtie [18] allowing at most two mismatches (controlled by option* -v 2*). Only the alignments in the best stratum are reported (if a read matches to six different genomic loci, two loci with no mismatch, and four loci with one mismatch, only the two alignments are reported) controlled with -*-strata* and -*-best*. Alignments for reads with more than 50 mapping locations are suppressed (*-k 50*). The mapped tags are annotated with the help of various models of coding and non-coding RNAs retrieved from the UCSC table browser [19]. Tags mapping to exonic regions of coding genes are excluded from further analysis. NcRNAs are quantified for each library independently. For tags mapping to multiple genomic loci the number of reads corresponding to the tag is divided by the number of mapping loci. To account for differences in sequencing depth, tag-counts are normalized (NEV, normalized expression value). Differential expression (corrected *P* value (FDR) < 0.1) is detected with the DESeq bioconductor package [20] that takes biological and technical variance into account.

To reduce noise we introduced an outlier detection prior to differential expression analysis into the omiRas pipeline. The normalized counts are evaluated by PCA with R 3.0.2. The samples identified to be four or more standard deviations away from the mean on the first or second principal component are considered outliers and are removed from analysis.

#### 2.3.2. Identification of miRNA Targets in ccRCC

MRNA targets (as given in the XLS file of Wozniak et al. [16]) of differentially expressed miRNAs were identified with the “interactive network tool” of omiRas. An interaction between an miRNA and a coding gene is assumed to be valid if the following two criteria apply.

- Three or more of seven miRNA-mRNA interaction databases support the interaction.
- The expression of the miRNA/mRNA pair is inverse. The miRNA is significantly downregulated and the mRNA is upregulated or* vice versa*.

#### 2.3.3. Identification of miRNAs Involved in the Deregulation of Genes from the Same Functional Category

Up- and downregulated genes in ccRCC were mapped to functional Gene Ontology (GO) categories using DAVID Bioinformatics Resources 6.7 [21]. Genes within enriched (FDR < 0.05) categories were committed to the STRING database [22] to determine protein-protein interactions of their gene products. Additionally, miRNAs that might be causative for the deregulation of genes within the category were detected as described above.

#### 2.3.4. Visualization

PCA and hierarchical clustering of the differentially expressed miRNAs were performed and visualized with R 3.0.2. Networks of genes from the same GO category were visualized with Cytoscape [23]. Visualizations of annotation statistics for each library were taken from omiRas.

## 3. Results

The samples of patient P 10 were identified as outliers (see methods) and consequently tissues from the patient were not considered for downstream analysis. Therefore, differences in the sncRNAs between 9 patients' kidney tumors and adjacent normal renal tissue have been assessed.

**Figure 1:**
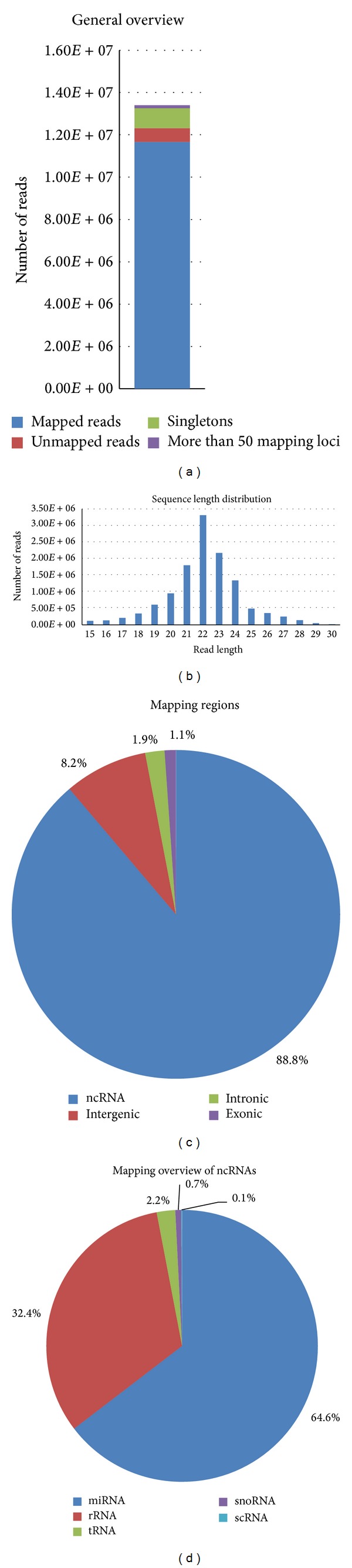
Annotation statistics for the control tissue of patient 1. (a) Statistics for the mapping of reads to the human genome. (b) Sequence length distribution after adapter clipping. (c) Mapping distribution for genomic regions. (d) Distribution of ncRNAs.

**Figure 2:**
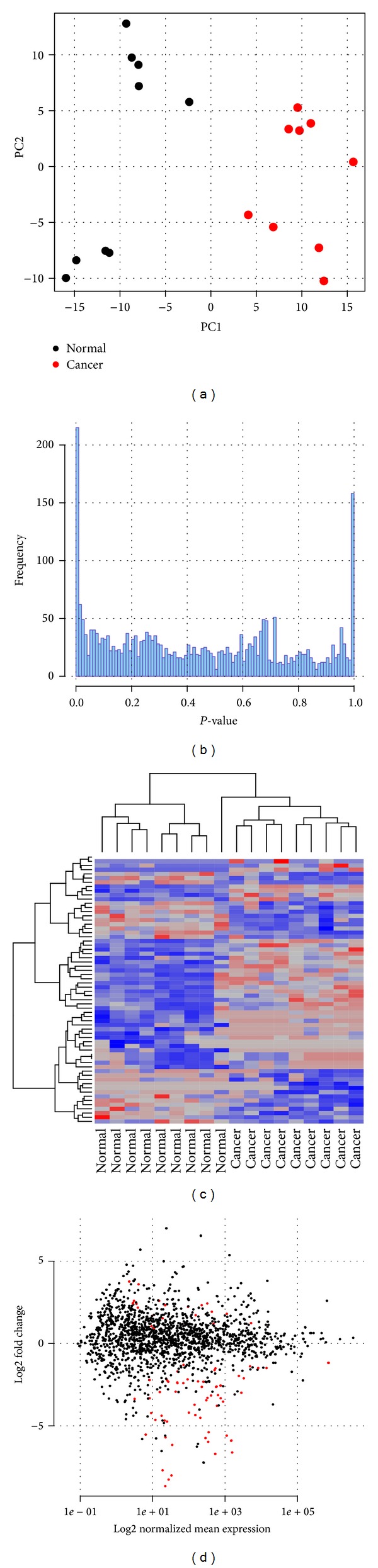
Potential of miRNAs to distinguish between normal and ccRCC tissue samples. (a) PCA of normalized expression values of miRNAs differentially expressed between conditions. The first principal component is given on the *X*-axis and the second on the *Y*-axis. (b) *P* value distribution of all miRNAs. (c) Unsupervised hierarchical cluster analysis of differentially expressed miRNAs. (d) An MA plot that visualizes the relation between log2 fold change between controls and cancer patients and the log2 average gene expression. MiRNAs with an FDR adjusted *P* value < 0.1 are indicated in red.

Overall, 306,958,418 sequences were processed. On average, 7.5% of reads were discarded during adapter clipping and quality trimming. The proportion of sncRNAs in the libraries ranged from 63% to 75%, whereas miRNAs accounted for 37–74% of sncRNAs (see Table 1). The length distribution after adapter clipping revealed a clear peak at 22 base pairs (bps) across all samples (Figure 1(b) (see Figure S1 in Supplementary Material available online at http://dx.doi.org/10.1155/2014/948408)), which is typical of mammalian miRNAs [24]. Besides ncRNA encoding loci reads were annotated to intergenic regions (Figure 1(c), S1) that may harbor novel miRNAs. The difference in miRNA proportions between libraries was attributed to varying degrees of snoRNAs, as well as tRNA derived fragments (Figure 1(d), S1). 1,672 sncRNAs were expressed in at least one library (Supplementary Table T1); 41 of these were significantly downregulated and 20 were upregulated in ccRCC tissues. The most differentially up-/downregulated miRNAs are listed in Table 2. The PCA of the normalized expression values of these miRNAs indicated a clear separation of normal and cancer tissue samples via the first two principle components (Figure 2(a)).

Similarly, an unsupervised two-dimensional hierarchical clustering of differentially expressed miRNAs clearly separated control and cancer samples (Figure 2(c)). An MA-plot (Figure 2(d)) shows the mean expression across libraries compared to the log2 fold change between conditions for all mature miRNAs. Significantly deregulated miRNAs are indicated in red. MiRNAs with high fold changes that are not differentially expressed exhibit a too high biological variance within the groups to reach the significance threshold. Many of the significantly deregulated miRNAs have already been implicated in ccRCC development, like the upregulated miR-106b-3p, 142-5p, 21-5p, 210, 361-3p, and miR-590 as well as the downregulated miR-10b-3p, 99a, 106a-5p, 135a-5p, 206, 363-3p, 500-3p, 508-5p, or miR-509-5p [25–28].

**Figure 3:**
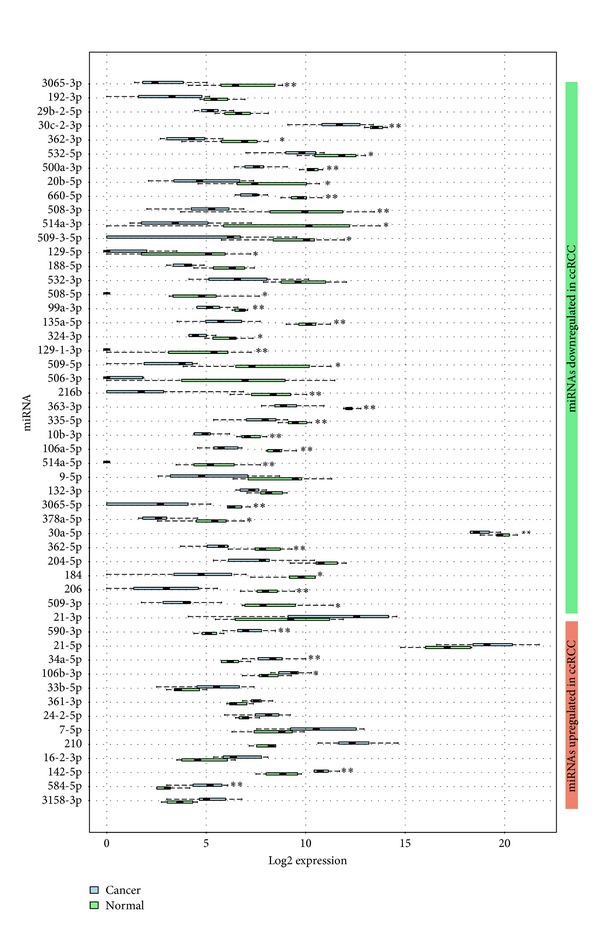
Boxplots of differentially expressed miRNAs in ccRCC. For each miRNA the distribution of NEVs for all samples from each condition is displayed adjacent to each other in log2 scale. Boxplots for the cancer group are visualized in blue and for the controls in green. **FDR < 0.01; *FDR < 0.05.

Other miRNAs and snoRNAs (downregulated, e.g.: miR-3065-5p, 660-5p, sno-HBII-85-25, upregulated: sno-ACA61, sno-ACA44, and miR-24) have not been discovered in this type of cancer until now. The expression of all significantly regulated miRNAs between normal and ccRCC tissues is given in Figure 3.

### 3.1. Detection of miRNA Targets and Functional Enrichment

**Figure 4:**
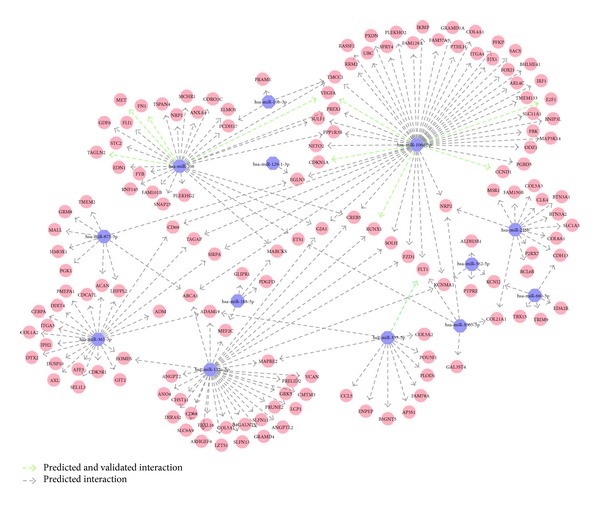
Interaction network of upregulated genes and downregulated miRNAs in ccRCC. Post-transcriptional regulation of a gene by an miRNA was assumed to be valid if it is supported by at least three interaction databases. MiRNAs are indicated in blue and mRNAs are indicated in red. Interactions between an miRNA and a 3′UTR of a gene are visualized by arrows. Green arrows visualize interactions that have recently been validated in cell lines; grey arrows indicate that the interaction currently lacks experimental validation.

To assert the influence of miRNAs on the gene expression pattern in ccRCC, we detected differentially expressed mRNAs with conserved seed sequences of inversely expressed miRNAs in their 3′UTR, supported by at least three miRNA-mRNA interaction databases. The network of upregulated genes and the 15 most significantly downregulated miRNAs is given in Figure 4. It is composed of 13 miRNAs and 144 mRNAs, where nine mRNAs (*NRP2, KCNMA1, FLT1, CREB5, EGLN3, ADAM19, ABAC1, MARCKS, *and* GJA1*) are under post-transcriptional control of three deregulated miRNAs. The miRNA with the largest number of targets in the network is miR-106a-5p that has seed sequences in ~1/3 of all mRNAs (49 targets). Other miRNAs with more than ten targets are miR-135a-5p (32 targets), miR-206 (28 targets), miR-363-3p (22 targets), and miR-216b (13 targets). Some of the predicted interactions in the network have recently been validated in different cell types, like the upregulation of the* c-Met* oncogene (*MET*) [29], fibronectin (*FN1*) [30], or vascular endothelial growth factor A (*VEGFA*) [31] due to loss of miR-206 or the upregulation of* E2F1 *[32],* CCND1 *[33], and* CDKN1A* [34] due to loss of miR-106a-5p. A similar analysis was performed for upregulated miRNAs and downregulated mRNAs and the interactions are given in Supplementary Table T2.

**Figure 5:**
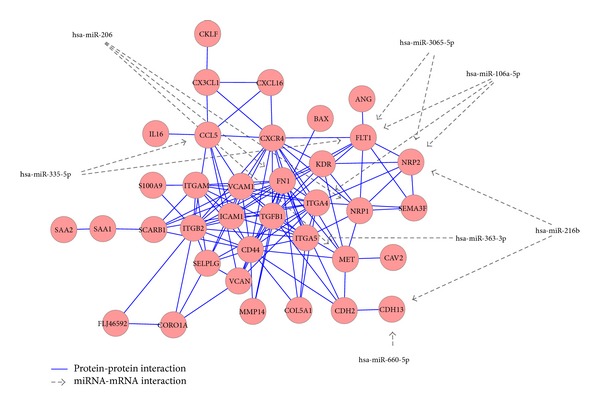
Influence of downregulated miRNAs on upgregulated genes from the GO category: “cell migration.” Interactions between gene products (red circles) are indicated by a blue line and interactions between an miRNA and an mRNA are indicated by a grey line.

In order to identify the influence of miRNAs on the metastatic potential of ccRCCs, we examined a subset of upregulated genes, enriched in GO category “cell motion” (FDR = 0.0002), and detected interactions of their gene products as well as the influence of miRNAs on the expression of the corresponding mRNAs. The interaction network is given in Figure 5. It is comprised of 36 mRNAs/gene products and six miRNAs. The network is centered around 12 highly connected gene/protein nodes, among these are transforming growth factor beta 1 (*TGFB1*), the vascular cell adhesion molecule-1 (*VCAM1*), the cell surface receptor CD44, and the intercellular adhesion molecule (*ICAM1*). The* NRP2* and* FLT1* mRNAs, both coding for* VEGFA* receptors, are potential targets of three and two miRNAs, respectively. Other targeted mRNAs from the network include cadherin 13 (*CDH13*), integrin A4 and A5 (*ITGA4*,* ITGA5*), chemokine* CCL5*, fibronectin (*FN1*), neuropilin-1 (*NRP1*), and the* MET* gene coding for the hepatocyte growth factor receptor.

## 4. Discussion

Our study links coding- and non-coding transcriptome data of normal and ccRCC tissue from two distinct studies. By the use of several miRNA-mRNA interaction databases available in omiRas we are able to provide new insights into the influence of aberrant miRNA expression on hundreds of deregulated genes.

Hypoxic regions of tumors are often resistant to both radiotherapy and chemotherapy [35] and under insufficient oxygen supply angiogenesis is stimulated through upregulation of* VEGF*. VEGF-mediated angiogenesis is thought to play a critical role in tumor growth and metastasis [36]. We show that miR-206 is among the most significantly downregulated miRNAs in ccRCC and miR-206 loss has likewise been reported under hypoxic conditions [15]. As given in Figure 3,* VEGFA* is one of the predicted targets of miR-206. This prediction has gained support by the study of Zhang et al., who showed that miR-206 downregulation promotes proliferation and invasion of laryngeal cancer by regulating* VEGF* expression [37].

In line with downregulation of miR-206 and upregualtion of* VEGFA* under hypoxia,* MET* overexpression can be caused by hypoxia [38]. The overexpression of the cell surface receptor Met tyrosine kinase MET is a hallmark of various types of cancers including pRCC and ccRCC [39]. In the normal kidney, ligand binding to MET mediates the activation of the MAPK, STAT, and a variety of other signaling pathways and promotes increased cell growth, scattering and motility, invasion, protection from apoptosis, branching morphogenesis, and angiogenesis [40].* MET* overexpression in transgenic mice led to spontaneous development of hepatocellular carcinoma and and inactivation of the* MET* gene to tumor regression [41]. We identified* MET* as a proposed target of miR-206 and this prediction is supported by Yan and colleagues [29], who showed that miR-206 targets* MET* and inhibits rhabdomyosarcoma development.

Fibronectin (encoded by* FN1*) is a multifunctional extracellular glycoprotein and its increased expression is significantly associated with a higher probability of metastasis, poorer overall survival, and distant metastasis development [42].* FN1* expression was significantly increased in hypoxic mouse embryonic stem cells [43] and downregulation of miR-206 has been shown to induce* FN1* upregulation in mouse lung tissues [30].

*NRP1* encodes a receptor for* VEGF* and a block to* NRP1* suppresses tumor growth due to decreased angiogenesis and cell proliferation [44]. In two different lung cancer cell lines, hypoxia regulated* NRP1* expression differently. In the A549 AC cell line the expression increased, whereas a decreased expression was reported in SKMES-1 SCC [45]. Regulation of VEGF as well as VEGF receptor gene expression has been ascribed to transcription factor* ETS1 *[46]. Oikawa et al. showed that hypoxia induced* ETS1* expression in human bladder cancer cell lines [47]. In several tumors, high* ETS1* expression has been associated with decreased survival, angiogenesis, and poor prognosis [46].

Myristoylated alanine-rich C kinase substrate (MARCKS) controls mucus granule secretion by airway epithelial cells and directed migration of leukocytes, stem cells, and fibroblasts. SiRNA knockdown of MARCKS expression in invasive lung cancer cell lines reduced migration [48]. In bladder cancer, MARCKS expression is induced under hypoxia [49].

Currently, an interaction between miR-206 and* NRP1*,* ETS1,* or* MARCKS* has not been experimentally verified but is predicted by our analysis. Therefore, these interactions represent an interesting target for experimental validation.

Taken together, loss of miR-206 under hypoxic conditions might be the reason for* VEGF*,* FN1, NRP1, ETS1,* and* MET* upregulation, all hallmark events of (ccRCC) carciogenesis. Notably, besides miR-206, downregulated miR-106a-5p has also been shown to interact with* VEGFA* [50]. Moreover, another gene coding for a* VEGFA* receptor (*FLT1*) is predicted to be regulated by miR-106a.

Other suggested targets of miR-106a by our analysis in ccRCC are* i.a. E2F1*,* CDKN1A,* and* PREX*. Upregulation of the transcription factor* E2F1, *a key regulator of proliferation and apoptosis, might be a driving force in the local and vascular infiltration of ccRCC [51]. In glioma, cell growth is inhibited by miR-106a due to post-transcriptional downregulation of* E2F1 *and accordingly downregulation of* p53* [32]. Campbell and colleagues [52] showed that a common predicted target of miR-206 and miR-106a,* PREX, *is essential for metastasis formation in several cancer types by influencing physical migration processes through effects upon Rac1-driven motility.

One miRNA without previous implications in ccRCC is miR-3065-5p, an antisense miRNA to miR-338 [53]. Due to its novelty, currently no experimentally validated interactions are known for this miRNA. Based on our predictions it might target the previously described* NRP2, FLT1, ETS1, *and* MARCKS *and its loss can thereby contribute to angiogenesis in ccRCC.

Taken together the analysis underlines the role of hypoxia as a key factor in kidney tumor angiogenesis [54], which might* i.a.* be regulated by the loss of miR-206. We show that miR-206 has several targets that are upregulated under hypoxic conditions. A couple of these predicted interactions have already been experimentally validated, which highlights the validity of our bioinformatical* in silico* approach.

## 5. Conclusion

In this study we demonstrate how the combined analysis of miRNA and mRNA data with omiRas can explain differential gene expression signatures via loss/gain of post-transcriptional control by deregulated miRNAs. Without the integration of coding gene expression, differentially expressed miRNAs can only serve as biomarkers with nonspecific function. We assign roles to miRNAs without laborious functional testing by concentrating on the “low hanging fruit,” namely, interactions between miRNAs and mRNAs with increased likelihood of interaction due to the support of several databases. These promising candidates can afterwards undergo further functional testing. The validity of this strategy is underlined by the fact that several predictions from our study have recently been validated in cell lines. This makes omiRas a beneficial tool in cancer research. Furthermore, omiRas is not only limited to cancer studies, but is a useful tool to integrate coding gene expression profiles into miRNA analysis for any dataset that compares two different biological conditions.

## Supplementary Material

T1: Mean normalized expression values for all sncRNAs in the control and the ccRCC libraries.
T2: Interactions between significantly upregulated miRNAs and downregulated mRNAs in ccRCC.
S1: Mapping statistics for all patient samples in each of the two conditions.

## Figures and Tables

**Table 1:** Overview of reads analyzed across nine samples of normal control (N) and ccRCC (C) tissues.

Library	Raw sequencing reads	After adapter and quality trimming	sncRNA	miRNA
N1	14,443,742	12,906,724	8,495,200	6,252,360
N2	14,307,074	13,331,072	10,356,100	6,854,590
N3	17,936,606	15,100,756	9,130,100	3,679,540
N4	17,343,176	16,241,449	10,934,800	5,447,920
N5	14,684,860	13,072,885	9,206,400	4,592,420
N6	18,696,751	17,718,335	13,328,400	8,738,310
N7	19,096,243	17,625,538	12,940,500	7,637,660
N8	18,754,729	17,405,763	12,780,900	5,327,410
N9	18,663,047	17,516,415	13,242,400	8,099,360

C1	14,324,200	13,188,486	10,027,000	7,155,830
C2	14,931,770	13,695,844	10,906,400	7,390,700
C3	17,643,505	16,762,300	13,248,300	8,391,030
C4	15,083,510	14,082,747	10,114,800	4,218,080
C5	14,057,127	13,045,222	9,260,080	4,099,150
C6	19,244,097	18,285,475	13,897,300	8,193,160
C7	18,889,231	17,589,421	13,477,500	7,093,640
C8	19,554,654	18,365,512	13,127,700	4,896,510
C9	19,304,096	18,173,426	13,676,700	6,011,960

Average	17,053,245.4	15,783,742.8	11,563,921.1	6,337,757.2

**Table 2:** Top 15 down-/upregulated sncRNAs in ccRCC tissues.

ncRNA	Control NEV	Cancer NEV	log2 Fold change	FDR
hsa-miR-363-3p	5,076.33	645,08	−2,98	1,2081*E* − 21
hsa-miR-500a-3p	1,289.69	221,65	−2,54	4,1147*E* − 19
hsa-miR-206	291,35	14,45	−4,33	6,3156*E* − 08
hsa-miR-10b-3p	153,08	34,08	−2,17	8,2369*E* − 08
hsa-miR-106a-5p	396,71	64,42	−2,62	2,8155*E* − 07
hsa-miR-135a-5p	1,127.62	82,86	−3,77	5,4484*E* − 07
hsa-miR-660-5p	981,73	158,15	−2,63	1,5614*E* − 06
hsa-miR-3065-5p	77,52	10,24	−2,92	3,8416*E* − 05
hsa-miR-30c-2-3p	12,274.78	4,399.26	−1,48	5,7416*E* − 05
hsa-miR-335-5p	781,07	258,25	−1,60	6,2893*E* − 05
hsa-miR-514a-5p	66,83	0,26	−8,01	6,3893*E* − 05
hsa-miR-129-1-3p	45,12	0,11	−8,64	0,00011999
hsa-miR-216b	402,11	17,60	−4,51	0,00012813
hsa-miR-188-5p	80,69	15,72	−2,36	0,0005944
hsa-miR-362-5p	302,80	62,05	−2,29	0,00087791

ncRNA	Control NEV	Cancer NEV	log2 Fold change	FDR

hsa-miR-584-5p	7,40	37,24	2,33	0,00060831
hsa-miR-590-3p	33,82	158,08	2,22	0,00074439
hsa-sno-ACA61	180,29	693,03	1,94	0,00115355
hsa-miR-142-5p	501,18	1,769.88	1,82	0,00136324
hsa-sno-mgh18S-121	47,08	111,93	1,25	0,00687682
hsa-miR-34a-5p	76,09	383,47	2,33	0,00921823
hsa-sno-ACA52	9,77	28,35	1,54	0,01048507
hsa-miR-106b-3p	294,41	680,87	1,21	0,02347042
hsa-miR-3182	4,33	26,20	2,60	0,03703154
hsa-sno-ACA44	54,31	201,32	1,89	0,05439511
hsa-miR-24-2-5p	127,79	292,76	1,20	0,06507243
hsa-miR-7-5p	439,97	3,243.48	2,88	0,06507243
hsa-miR-210	1,062.71	7,973.01	2,91	0,06507243
hsa-miR-16-2-3p	35,85	123,42	1,78	0,06507243

NEV: normalized expression value; Log2 Fold change: log2[cancer/control ratio]; FDR: corrected *P* value.
